# Supplementary material for: Hot spot prediction in protein-protein interactions by an ensemble system
Source: BMC Syst Biol. 2018 Dec 31;12(Suppl 9):132. doi: 10.1186/s12918-018-0665-8 (PMC6311905; doi:10.1186/s12918-018-0665-8)
Supplement: Supplementary file 2 — All relevant MATLAB code. A simple Matlab implement of our predictor. (ZIP 19,742 kb) [file 12918_2018_665_MOESM2_ESM.zip › Additional file 2/Read ASA/ASA website/dbMPIKT/4PWXA.html]

 NetSurfP - Protein Surface Accessibility and Secondary Structure Predictions

  

|  |  |  |
| --- | --- | --- |
|  |  | NetSurfP - Protein Surface Accessibility and Secondary Structure Predictions  Technical University of Denmark |

  


---

```
# For publication of results, please cite:
# A generic method for assignment of reliability scores applied to solvent accessibility predictions.
# Bent Petersen, Thomas Nordahl Petersen, Pernille Andersen, Morten Nielsen and Claus Lundegaard
# BMC Structural Biology 2009, 9:51 doi:10.1186/1472-6807-9-51
#
# Column 1: Class assignment - B for buried or E for Exposed - Threshold: 25% exposure, but not based on RSA
# Column 2: Amino acid
# Column 3: Sequence name
# Column 4: Amino acid number
# Column 5: Relative Surface Accessibility - RSA
# Column 6: Absolute Surface Accessibility
# Column 7: Z-fit score for RSA prediction
# Column 8: Probability for Alpha-Helix
# Column 9: Probability for Beta-strand
# Column 10: Probability for Coil
E S  4PWXA                  1    0.832  97.569  -0.166   0.003   0.003   0.994
E V  4PWXA                  2    0.452  69.503  -1.219   0.058   0.017   0.925
E D  4PWXA                  3    0.704 101.461  -0.607   0.115   0.016   0.868
E D  4PWXA                  4    0.620  89.356  -1.284   0.115   0.016   0.868
E L  4PWXA                  5    0.345  63.133  -0.737   0.115   0.016   0.868
E T  4PWXA                  6    0.578  80.224   0.154   0.058   0.017   0.925
B V  4PWXA                  7    0.230  35.320   0.132   0.115   0.016   0.868
E E  4PWXA                  8    0.526  91.927   0.711   0.058   0.017   0.925
E P  4PWXA                  9    0.519  73.674  -0.573   0.181   0.016   0.803
E N  4PWXA                 10    0.564  82.526   0.745   0.181   0.016   0.803
B L  4PWXA                 11    0.088  16.186   0.225   0.430   0.016   0.555
E H  4PWXA                 12    0.626 113.833   1.263   0.430   0.016   0.555
E S  4PWXA                 13    0.543  63.593   0.489   0.430   0.016   0.555
B L  4PWXA                 14    0.196  35.814   0.064   0.430   0.016   0.555
B I  4PWXA                 15    0.213  39.386  -1.121   0.257   0.016   0.727
E T  4PWXA                 16    0.698  96.799   0.616   0.257   0.016   0.727
E S  4PWXA                 17    0.630  73.883  -0.708   0.115   0.016   0.868
E T  4PWXA                 18    0.556  77.076   0.297   0.058   0.017   0.925
E T  4PWXA                 19    0.278  38.489   0.176   0.005   0.045   0.951
B H  4PWXA                 20    0.160  29.104   1.056   0.004   0.420   0.576
B K  4PWXA                 21    0.125  25.733   1.157   0.001   0.900   0.099
B W  4PWXA                 22    0.024   5.724   1.467   0.000   0.983   0.017
B I  4PWXA                 23    0.014   2.627   1.859   0.000   0.983   0.017
B F  4PWXA                 24    0.015   3.011   1.575   0.000   0.983   0.017
B V  4PWXA                 25    0.018   2.782   1.337   0.001   0.959   0.040
B G  4PWXA                 26    0.033   2.636  -0.527   0.002   0.816   0.182
B G  4PWXA                 27    0.147  11.608  -0.595   0.004   0.197   0.799
B K  4PWXA                 28    0.232  47.722  -0.223   0.005   0.045   0.951
B G  4PWXA                 29    0.194  15.299  -1.617   0.018   0.019   0.964
B G  4PWXA                 30    0.177  13.906  -1.934   0.053   0.043   0.903
B V  4PWXA                 31    0.074  11.405  -1.188   0.113   0.087   0.800
B G  4PWXA                 32    0.050   3.951  -1.005   0.278   0.093   0.628
B K  4PWXA                 33    0.067  13.802   0.357   0.717   0.014   0.269
B T  4PWXA                 34    0.051   7.074  -0.089   0.879   0.010   0.111
B T  4PWXA                 35    0.057   7.864   0.275   0.938   0.007   0.055
B S  4PWXA                 36    0.035   4.090   0.281   0.975   0.003   0.022
B S  4PWXA                 37    0.017   1.992   1.077   0.970   0.001   0.030
B C  4PWXA                 38    0.029   4.072   0.065   0.970   0.001   0.030
B S  4PWXA                 39    0.045   5.262  -0.350   0.970   0.001   0.030
B I  4PWXA                 40    0.036   6.734   0.221   0.970   0.001   0.030
B A  4PWXA                 41    0.020   2.204   0.494   0.970   0.001   0.030
B I  4PWXA                 42    0.084  15.540   0.209   0.970   0.001   0.030
B Q  4PWXA                 43    0.070  12.448  -0.163   0.970   0.001   0.030
B M  4PWXA                 44    0.054  10.905   0.773   0.970   0.001   0.030
B A  4PWXA                 45    0.046   5.036  -0.422   0.970   0.001   0.030
B L  4PWXA                 46    0.214  39.128   0.145   0.923   0.002   0.076
E S  4PWXA                 47    0.313  36.672  -0.208   0.782   0.003   0.216
E Q  4PWXA                 48    0.509  90.979  -0.380   0.502   0.002   0.495
E P  4PWXA                 49    0.520  73.731   0.270   0.053   0.005   0.942
E N  4PWXA                 50    0.673  98.483  -0.553   0.003   0.003   0.994
B K  4PWXA                 51    0.166  34.084   1.324   0.005   0.045   0.951
E Q  4PWXA                 52    0.265  47.347   1.376   0.004   0.514   0.481
B F  4PWXA                 53    0.022   4.375   1.242   0.001   0.959   0.040
B L  4PWXA                 54    0.014   2.563   1.757   0.000   0.983   0.017
B L  4PWXA                 55    0.017   3.149   1.313   0.000   0.983   0.017
B I  4PWXA                 56    0.018   3.312   1.372   0.001   0.959   0.040
B S  4PWXA                 57    0.030   3.551  -0.631   0.002   0.816   0.182
B T  4PWXA                 58    0.041   5.673  -0.057   0.005   0.262   0.733
B V  4PWXA                 59    0.098  15.078   0.463   0.005   0.045   0.951
B P  4PWXA                 60    0.207  29.345  -0.671   0.018   0.019   0.964
B A  4PWXA                 61    0.206  22.712  -0.890   0.016   0.005   0.979
B H  4PWXA                 62    0.161  29.231  -1.162   0.053   0.005   0.942
B N  4PWXA                 63    0.151  22.092  -1.320   0.053   0.005   0.942
B L  4PWXA                 64    0.031   5.621   0.181   0.600   0.003   0.397
B S  4PWXA                 65    0.143  16.795  -0.684   0.622   0.015   0.363
E D  4PWXA                 66    0.403  58.000  -0.481   0.717   0.014   0.269
B A  4PWXA                 67    0.104  11.472  -1.597   0.717   0.014   0.269
B F  4PWXA                 68    0.077  15.414  -0.071   0.561   0.047   0.393
E G  4PWXA                 69    0.504  39.704  -0.757   0.115   0.016   0.868
B E  4PWXA                 70    0.194  33.892   0.711   0.053   0.043   0.903
E K  4PWXA                 71    0.535 110.008   0.088   0.056   0.142   0.802
B F  4PWXA                 72    0.275  55.213  -0.524   0.052   0.084   0.864
E G  4PWXA                 73    0.562  44.222  -1.109   0.053   0.043   0.903
E K  4PWXA                 74    0.615 126.588  -1.644   0.053   0.043   0.903
E D  4PWXA                 75    0.532  76.632  -0.296   0.053   0.043   0.903
E A  4PWXA                 76    0.300  33.093  -1.339   0.113   0.087   0.800
E R  4PWXA                 77    0.498 113.950  -0.280   0.125   0.227   0.648
E K  4PWXA                 78    0.484  99.621   0.125   0.135   0.317   0.548
B V  4PWXA                 79    0.174  26.775  -1.060   0.135   0.317   0.548
E T  4PWXA                 80    0.542  75.162  -1.309   0.125   0.227   0.648
E G  4PWXA                 81    0.432  33.975  -1.231   0.118   0.150   0.732
B M  4PWXA                 82    0.238  47.704  -0.715   0.118   0.150   0.732
E N  4PWXA                 83    0.566  82.818  -1.053   0.113   0.087   0.800
E N  4PWXA                 84    0.445  65.148  -0.715   0.191   0.086   0.723
B L  4PWXA                 85    0.155  28.344  -0.482   0.199   0.152   0.649
B S  4PWXA                 86    0.246  28.808  -0.331   0.216   0.235   0.548
B C  4PWXA                 87    0.152  21.355  -1.453   0.231   0.330   0.439
B M  4PWXA                 88    0.220  44.102  -0.514   0.231   0.330   0.439
E E  4PWXA                 89    0.348  60.796  -0.933   0.135   0.317   0.548
B I  4PWXA                 90    0.114  21.164  -0.621   0.125   0.227   0.648
E D  4PWXA                 91    0.341  49.138  -0.326   0.113   0.087   0.800
B P  4PWXA                 92    0.218  30.934  -0.699   0.522   0.016   0.462
E S  4PWXA                 93    0.530  62.116  -0.308   0.717   0.014   0.269
E A  4PWXA                 94    0.405  44.642   0.123   0.802   0.014   0.185
B A  4PWXA                 95    0.142  15.692  -0.860   0.802   0.014   0.185
B L  4PWXA                 96    0.107  19.628  -0.741   0.802   0.014   0.185
E K  4PWXA                 97    0.478  98.345   0.243   0.802   0.014   0.185
E D  4PWXA                 98    0.494  71.142   0.071   0.802   0.014   0.185
B M  4PWXA                 99    0.106  21.111  -0.173   0.802   0.014   0.185
B N  4PWXA                100    0.205  29.953   0.011   0.879   0.010   0.111
E D  4PWXA                101    0.540  77.756  -0.596   0.802   0.014   0.185
E M  4PWXA                102    0.445  89.004  -0.117   0.802   0.014   0.185
B A  4PWXA                103    0.087   9.587  -0.566   0.717   0.014   0.269
B V  4PWXA                104    0.214  32.953  -0.468   0.660   0.049   0.291
E S  4PWXA                105    0.476  55.764  -0.303   0.561   0.047   0.393
E R  4PWXA                106    0.421  96.363  -0.373   0.561   0.047   0.393
B A  4PWXA                107    0.142  15.659  -1.527   0.561   0.047   0.393
E N  4PWXA                108    0.347  50.874  -0.537   0.561   0.047   0.393
E N  4PWXA                109    0.438  64.109  -1.428   0.455   0.046   0.498
E N  4PWXA                110    0.481  70.374  -1.421   0.339   0.016   0.645
B G  4PWXA                111    0.201  15.858  -1.613   0.354   0.048   0.598
E S  4PWXA                112    0.300  35.101  -0.800   0.268   0.043   0.689
E D  4PWXA                113    0.496  71.517  -0.984   0.354   0.048   0.598
B G  4PWXA                114    0.169  13.316  -1.676   0.354   0.048   0.598
B Q  4PWXA                115    0.182  32.523  -1.117   0.502   0.102   0.396
B G  4PWXA                116    0.191  15.016  -1.166   0.455   0.046   0.498
E D  4PWXA                117    0.373  53.720  -1.291   0.455   0.046   0.498
B D  4PWXA                118    0.177  25.477  -1.506   0.455   0.046   0.498
B L  4PWXA                119    0.114  20.947  -1.130   0.455   0.046   0.498
E G  4PWXA                120    0.343  26.978  -0.744   0.455   0.046   0.498
E S  4PWXA                121    0.354  41.430  -1.401   0.455   0.046   0.498
B L  4PWXA                122    0.271  49.620  -1.404   0.430   0.016   0.555
B L  4PWXA                123    0.265  48.576  -1.816   0.339   0.016   0.645
E Q  4PWXA                124    0.347  61.921  -1.667   0.257   0.016   0.727
E G  4PWXA                125    0.412  32.409  -1.956   0.257   0.016   0.727
E G  4PWXA                126    0.363  28.592  -1.503   0.522   0.016   0.462
B A  4PWXA                127    0.254  27.980  -1.025   0.622   0.015   0.363
B L  4PWXA                128    0.122  22.247  -0.598   0.717   0.014   0.269
E A  4PWXA                129    0.293  32.278  -0.063   0.802   0.014   0.185
E D  4PWXA                130    0.443  63.851  -0.618   0.802   0.014   0.185
B L  4PWXA                131    0.157  28.692  -1.067   0.694   0.003   0.303
B T  4PWXA                132    0.208  28.891  -1.212   0.600   0.003   0.397
E G  4PWXA                133    0.486  38.217  -0.771   0.502   0.002   0.495
E S  4PWXA                134    0.490  57.475  -1.415   0.321   0.003   0.675
B I  4PWXA                135    0.181  33.522   0.044   0.176   0.004   0.820
E P  4PWXA                136    0.398  56.405  -1.224   0.176   0.004   0.820
E G  4PWXA                137    0.396  31.157  -1.005   0.246   0.004   0.750
B I  4PWXA                138    0.152  28.138  -0.405   0.694   0.003   0.303
E D  4PWXA                139    0.447  64.427  -0.236   0.782   0.003   0.216
E E  4PWXA                140    0.327  57.197  -0.607   0.858   0.002   0.139
B A  4PWXA                141    0.159  17.467  -0.980   0.858   0.002   0.139
B L  4PWXA                142    0.194  35.503  -0.934   0.782   0.003   0.216
B S  4PWXA                143    0.215  25.175  -0.947   0.782   0.003   0.216
B F  4PWXA                144    0.168  33.637  -0.682   0.923   0.002   0.076
E M  4PWXA                145    0.329  65.873  -0.756   0.923   0.002   0.076
E E  4PWXA                146    0.319  55.712  -0.041   0.923   0.002   0.076
B V  4PWXA                147    0.071  10.943  -1.147   0.970   0.001   0.030
B M  4PWXA                148    0.203  40.540  -0.126   0.970   0.001   0.030
E K  4PWXA                149    0.450  92.565   0.650   0.970   0.001   0.030
B H  4PWXA                150    0.118  21.428   0.274   0.970   0.001   0.030
B I  4PWXA                151    0.048   8.843  -0.327   0.970   0.001   0.030
E K  4PWXA                152    0.409  84.152   0.886   0.923   0.002   0.076
E R  4PWXA                153    0.506 115.782   1.029   0.923   0.002   0.076
B Q  4PWXA                154    0.093  16.538   0.086   0.782   0.003   0.216
B E  4PWXA                155    0.196  34.259  -0.366   0.694   0.003   0.303
E Q  4PWXA                156    0.576 102.874   0.035   0.694   0.003   0.303
E G  4PWXA                157    0.571  44.922   0.153   0.600   0.003   0.397
B E  4PWXA                158    0.183  32.057  -0.333   0.502   0.002   0.495
E G  4PWXA                159    0.432  34.014  -0.268   0.321   0.003   0.675
E E  4PWXA                160    0.655 114.516  -0.323   0.058   0.017   0.925
E T  4PWXA                161    0.497  68.976   0.928   0.018   0.019   0.964
B F  4PWXA                162    0.048   9.593   1.140   0.004   0.085   0.910
B D  4PWXA                163    0.202  29.137   0.222   0.005   0.262   0.733
B T  4PWXA                164    0.052   7.254   1.190   0.001   0.959   0.040
B V  4PWXA                165    0.012   1.860   1.929   0.000   0.983   0.017
B I  4PWXA                166    0.015   2.812   1.827   0.000   0.983   0.017
B F  4PWXA                167    0.034   6.804   0.225   0.001   0.959   0.040
B D  4PWXA                168    0.065   9.395   0.123   0.004   0.514   0.481
B T  4PWXA                169    0.054   7.518   0.317   0.004   0.085   0.910
B A  4PWXA                170    0.198  21.875  -0.416   0.005   0.015   0.979
B P  4PWXA                171    0.099  14.076  -1.882   0.016   0.005   0.979
B T  4PWXA                172    0.202  28.045  -1.034   0.246   0.004   0.750
E G  4PWXA                173    0.287  22.579  -1.566   0.502   0.002   0.495
E H  4PWXA                174    0.303  55.116  -1.542   0.502   0.002   0.495
B T  4PWXA                175    0.073  10.153  -0.852   0.600   0.003   0.397
B L  4PWXA                176    0.077  14.080  -0.768   0.717   0.014   0.269
E R  4PWXA                177    0.282  64.486   0.317   0.717   0.014   0.269
B F  4PWXA                178    0.096  19.347  -0.396   0.717   0.014   0.269
B L  4PWXA                179    0.086  15.747  -0.379   0.522   0.016   0.462
B Q  4PWXA                180    0.251  44.793  -0.729   0.522   0.016   0.462
B L  4PWXA                181    0.079  14.392  -0.006   0.717   0.014   0.269
B P  4PWXA                182    0.156  22.193   0.100   0.858   0.002   0.139
E N  4PWXA                183    0.400  58.589  -0.454   0.923   0.002   0.076
B T  4PWXA                184    0.168  23.329  -0.527   0.970   0.001   0.030
B L  4PWXA                185    0.067  12.304  -0.248   0.970   0.001   0.030
B S  4PWXA                186    0.206  24.155  -0.453   0.970   0.001   0.030
E K  4PWXA                187    0.341  70.164  -0.104   0.970   0.001   0.030
B L  4PWXA                188    0.083  15.124  -0.174   0.970   0.001   0.030
B L  4PWXA                189    0.086  15.802  -0.102   0.923   0.002   0.076
E E  4PWXA                190    0.419  73.269  -0.206   0.923   0.002   0.076
B K  4PWXA                191    0.214  44.102  -0.307   0.923   0.002   0.076
B F  4PWXA                192    0.052  10.497  -0.321   0.923   0.002   0.076
B G  4PWXA                193    0.194  15.268   0.183   0.923   0.002   0.076
E E  4PWXA                194    0.385  67.242   0.125   0.923   0.002   0.076
B I  4PWXA                195    0.061  11.266  -1.026   0.923   0.002   0.076
B T  4PWXA                196    0.171  23.745  -0.689   0.923   0.002   0.076
E N  4PWXA                197    0.444  65.002  -0.390   0.923   0.002   0.076
B K  4PWXA                198    0.237  48.751   0.295   0.923   0.002   0.076
B L  4PWXA                199    0.041   7.434  -0.986   0.879   0.010   0.111
B G  4PWXA                200    0.246  19.321   0.039   0.879   0.010   0.111
E P  4PWXA                201    0.455  64.550   0.245   0.802   0.014   0.185
B M  4PWXA                202    0.112  22.391  -1.342   0.782   0.003   0.216
B L  4PWXA                203    0.188  34.386  -0.642   0.694   0.003   0.303
E N  4PWXA                204    0.520  76.099  -0.544   0.694   0.003   0.303
E S  4PWXA                205    0.380  44.524  -0.800   0.600   0.003   0.397
B F  4PWXA                206    0.192  38.635  -1.750   0.600   0.003   0.397
E M  4PWXA                207    0.348  69.575  -1.062   0.502   0.002   0.495
E G  4PWXA                208    0.507  39.862  -1.183   0.430   0.016   0.555
E A  4PWXA                209    0.334  36.807  -1.787   0.321   0.003   0.675
E G  4PWXA                210    0.348  27.364  -1.660   0.176   0.004   0.820
E N  4PWXA                211    0.436  63.830  -1.037   0.339   0.016   0.645
E V  4PWXA                212    0.444  68.243  -0.577   0.802   0.014   0.185
E D  4PWXA                213    0.465  67.021   0.233   0.879   0.010   0.111
B I  4PWXA                214    0.072  13.412  -1.049   0.923   0.002   0.076
B S  4PWXA                215    0.137  16.045   0.210   0.970   0.001   0.030
E G  4PWXA                216    0.493  38.823   0.701   0.970   0.001   0.030
B K  4PWXA                217    0.201  41.428   0.634   0.970   0.001   0.030
B L  4PWXA                218    0.034   6.299  -0.286   0.970   0.001   0.030
E N  4PWXA                219    0.372  54.402   0.783   0.970   0.001   0.030
E E  4PWXA                220    0.501  87.595   1.579   0.970   0.001   0.030
B L  4PWXA                221    0.058  10.620   0.726   0.970   0.001   0.030
B K  4PWXA                222    0.212  43.670   0.876   0.970   0.001   0.030
E A  4PWXA                223    0.546  60.125   1.299   0.970   0.001   0.030
B N  4PWXA                224    0.209  30.524   1.667   0.970   0.001   0.030
B V  4PWXA                225    0.024   3.643   0.748   0.988   0.000   0.012
E E  4PWXA                226    0.510  89.080   1.786   0.988   0.000   0.012
E T  4PWXA                227    0.397  55.119   1.890   0.988   0.000   0.012
B I  4PWXA                228    0.022   3.996   0.655   0.988   0.000   0.012
B R  4PWXA                229    0.248  56.792   1.291   0.970   0.001   0.030
E Q  4PWXA                230    0.623 111.196   1.515   0.975   0.003   0.022
B Q  4PWXA                231    0.183  32.773   1.330   0.923   0.002   0.076
B F  4PWXA                232    0.054  10.918   0.195   0.782   0.003   0.216
E T  4PWXA                233    0.518  71.902   0.691   0.406   0.004   0.590
E D  4PWXA                234    0.306  44.095   0.014   0.053   0.005   0.942
E P  4PWXA                235    0.509  72.227  -0.224   0.058   0.017   0.925
E D  4PWXA                236    0.580  83.549  -0.334   0.018   0.047   0.935
E L  4PWXA                237    0.267  48.906   1.062   0.004   0.197   0.799
B T  4PWXA                238    0.082  11.415  -0.456   0.004   0.420   0.576
B T  4PWXA                239    0.090  12.538  -0.066   0.001   0.900   0.099
B F  4PWXA                240    0.026   5.258   0.201   0.001   0.959   0.040
B V  4PWXA                241    0.052   7.962   0.822   0.001   0.959   0.040
B C  4PWXA                242    0.026   3.580   0.498   0.001   0.959   0.040
B V  4PWXA                243    0.034   5.180  -0.174   0.001   0.959   0.040
B C  4PWXA                244    0.024   3.398   0.324   0.003   0.718   0.279
B I  4PWXA                245    0.147  27.121   0.918   0.004   0.197   0.799
B S  4PWXA                246    0.125  14.615  -0.614   0.053   0.043   0.903
E E  4PWXA                247    0.392  68.413   0.246   0.181   0.016   0.803
E F  4PWXA                248    0.503 100.872  -0.573   0.257   0.016   0.727
B L  4PWXA                249    0.170  31.054  -0.313   0.257   0.016   0.727
B S  4PWXA                250    0.153  17.908  -0.547   0.339   0.016   0.645
E L  4PWXA                251    0.280  51.323   0.076   0.858   0.002   0.139
E Y  4PWXA                252    0.417  89.113   0.812   0.970   0.001   0.030
B E  4PWXA                253    0.172  30.083   0.534   0.970   0.001   0.030
B T  4PWXA                254    0.036   4.952   0.045   0.970   0.001   0.030
E E  4PWXA                255    0.376  65.617   1.141   0.970   0.001   0.030
E R  4PWXA                256    0.338  77.333   1.605   0.970   0.001   0.030
B L  4PWXA                257    0.027   4.925   0.133   0.970   0.001   0.030
B I  4PWXA                258    0.140  25.974   0.974   0.970   0.001   0.030
E Q  4PWXA                259    0.519  92.711   1.374   0.970   0.001   0.030
B E  4PWXA                260    0.178  31.079   0.199   0.970   0.001   0.030
B L  4PWXA                261    0.045   8.148   0.047   0.970   0.001   0.030
E I  4PWXA                262    0.527  97.550   1.268   0.923   0.002   0.076
E S  4PWXA                263    0.684  80.177   0.725   0.782   0.003   0.216
E Y  4PWXA                264    0.334  71.290   0.201   0.406   0.004   0.590
E D  4PWXA                265    0.734 105.798  -1.569   0.016   0.005   0.979
B M  4PWXA                266    0.218  43.642  -0.220   0.018   0.047   0.935
E D  4PWXA                267    0.534  76.906   0.354   0.019   0.141   0.840
B V  4PWXA                268    0.109  16.799   0.308   0.022   0.359   0.619
B N  4PWXA                269    0.247  36.205  -0.657   0.022   0.359   0.619
E S  4PWXA                270    0.258  30.273  -0.041   0.023   0.655   0.322
B I  4PWXA                271    0.066  12.247  -0.383   0.018   0.846   0.136
B I  4PWXA                272    0.168  31.043  -0.006   0.021   0.756   0.223
B V  4PWXA                273    0.076  11.666  -0.748   0.022   0.552   0.426
B N  4PWXA                274    0.157  22.941  -1.306   0.021   0.279   0.699
B Q  4PWXA                275    0.232  41.417  -1.796   0.020   0.205   0.775
B L  4PWXA                276    0.179  32.793  -2.255   0.021   0.279   0.699
B L  4PWXA                277    0.176  32.152   0.136   0.022   0.359   0.619
E F  4PWXA                278    0.333  66.773  -0.317   0.020   0.205   0.775
E A  4PWXA                279    0.294  32.344  -0.992   0.052   0.084   0.864
E E  4PWXA                280    0.661 115.424  -1.277   0.053   0.043   0.903
E N  4PWXA                281    0.399  58.472  -1.004   0.058   0.017   0.925
E D  4PWXA                282    0.278  40.002  -0.826   0.018   0.019   0.964
E Q  4PWXA                283    0.455  81.334  -0.937   0.176   0.004   0.820
E E  4PWXA                284    0.652 113.852  -0.849   0.321   0.003   0.675
E H  4PWXA                285    0.428  77.890  -0.892   0.321   0.003   0.675
B N  4PWXA                286    0.281  41.080  -0.999   0.406   0.004   0.590
B C  4PWXA                287    0.185  25.988  -0.338   0.406   0.004   0.590
E K  4PWXA                288    0.577 118.607   0.963   0.694   0.003   0.303
B R  4PWXA                289    0.199  45.594   0.258   0.782   0.003   0.216
B C  4PWXA                290    0.062   8.677   0.179   0.858   0.002   0.139
E Q  4PWXA                291    0.502  89.675   0.830   0.923   0.002   0.076
E A  4PWXA                292    0.442  48.675  -0.323   0.923   0.002   0.076
B R  4PWXA                293    0.187  42.709   0.656   0.923   0.002   0.076
B W  4PWXA                294    0.203  48.701   0.767   0.923   0.002   0.076
E K  4PWXA                295    0.653 134.404   1.326   0.970   0.001   0.030
E M  4PWXA                296    0.341  68.254   0.705   0.923   0.002   0.076
B Q  4PWXA                297    0.085  15.110  -0.668   0.923   0.002   0.076
E K  4PWXA                298    0.471  96.885   1.132   0.970   0.001   0.030
E K  4PWXA                299    0.573 117.866   1.715   0.970   0.001   0.030
B Y  4PWXA                300    0.112  23.849   0.860   0.970   0.001   0.030
B L  4PWXA                301    0.093  16.955   0.931   0.970   0.001   0.030
E D  4PWXA                302    0.603  86.950   1.656   0.970   0.001   0.030
E Q  4PWXA                303    0.316  56.473   1.866   0.970   0.001   0.030
B I  4PWXA                304    0.024   4.422   1.358   0.970   0.001   0.030
E D  4PWXA                305    0.409  58.879   1.777   0.923   0.002   0.076
E E  4PWXA                306    0.635 110.900   1.084   0.858   0.002   0.139
B L  4PWXA                307    0.184  33.654   0.172   0.782   0.003   0.216
B Y  4PWXA                308    0.063  13.484   0.146   0.502   0.002   0.495
E E  4PWXA                309    0.667 116.595   1.320   0.246   0.004   0.750
E D  4PWXA                310    0.716 103.176   0.357   0.053   0.005   0.942
B F  4PWXA                311    0.138  27.636   1.340   0.005   0.045   0.951
E H  4PWXA                312    0.465  84.602   0.590   0.004   0.197   0.799
B V  4PWXA                313    0.184  28.281   0.330   0.002   0.816   0.182
B V  4PWXA                314    0.160  24.531   0.678   0.001   0.900   0.099
E K  4PWXA                315    0.339  69.712   1.276   0.001   0.900   0.099
B M  4PWXA                316    0.036   7.184   0.872   0.004   0.616   0.381
B P  4PWXA                317    0.246  34.851   0.048   0.005   0.336   0.660
B L  4PWXA                318    0.198  36.290  -0.368   0.019   0.141   0.840
B C  4PWXA                319    0.222  31.113   0.967   0.018   0.088   0.893
E A  4PWXA                320    0.614  67.718   0.084   0.018   0.047   0.935
E G  4PWXA                321    0.575  45.245  -0.632   0.018   0.019   0.964
E E  4PWXA                322    0.574 100.190  -0.121   0.018   0.019   0.964
B I  4PWXA                323    0.172  31.838  -1.391   0.053   0.043   0.903
E R  4PWXA                324    0.349  79.898   0.041   0.053   0.043   0.903
E G  4PWXA                325    0.268  21.099  -0.103   0.058   0.017   0.925
B L  4PWXA                326    0.184  33.764   1.165   0.782   0.003   0.216
E N  4PWXA                327    0.641  93.872   0.474   0.858   0.002   0.139
B N  4PWXA                328    0.216  31.622   1.164   0.923   0.002   0.076
B L  4PWXA                329    0.018   3.223   1.435   0.970   0.001   0.030
E T  4PWXA                330    0.334  46.256   1.595   0.970   0.001   0.030
E K  4PWXA                331    0.484  99.600   2.079   0.970   0.001   0.030
B F  4PWXA                332    0.039   7.928   0.734   0.970   0.001   0.030
B S  4PWXA                333    0.035   4.102  -0.569   0.970   0.001   0.030
E Q  4PWXA                334    0.593 105.946   1.524   0.923   0.002   0.076
E F  4PWXA                335    0.343  68.740   1.281   0.858   0.002   0.139
B L  4PWXA                336    0.039   7.178  -0.084   0.694   0.003   0.303
B N  4PWXA                337    0.268  39.191   0.265   0.406   0.004   0.590
E K  4PWXA                338    0.604 124.181   0.445   0.109   0.005   0.886
E E  4PWXA                339    0.647 113.066  -1.264   0.053   0.005   0.942
E Y  4PWXA                340    0.534 114.116  -0.560   0.018   0.019   0.964
E N  4PWXA                341    0.429  62.806  -0.717   0.018   0.019   0.964
B P  4PWXA                342    0.220  31.147  -0.651   0.058   0.017   0.925
B I  4PWXA                343    0.203  37.499  -0.416   0.113   0.043   0.844
E T  4PWXA                344    0.493  68.365  -0.851   0.113   0.043   0.844
E D  4PWXA                345    0.451  64.989  -1.181   0.181   0.016   0.803
E G  4PWXA                346    0.364  28.686  -1.088   0.181   0.016   0.803
E K  4PWXA                347    0.542 111.510   0.598   0.268   0.043   0.689
B V  4PWXA                348    0.142  21.810   0.504   0.278   0.093   0.628
B I  4PWXA                349    0.110  20.313  -0.320   0.278   0.093   0.628
E Y  4PWXA                350    0.366  78.171   0.386   0.386   0.097   0.517
E E  4PWXA                351    0.508  88.748   0.919   0.386   0.097   0.517
B L  4PWXA                352    0.101  18.493   0.231   0.455   0.046   0.498
E E  4PWXA                353    0.454  79.296   0.644   0.455   0.046   0.498
E D  4PWXA                354    0.674  97.181   1.039   0.354   0.048   0.598
E K  4PWXA                355    0.456  93.799  -0.667   0.181   0.016   0.803
E E  4PWXA                356    0.872 152.408   0.814   0.003   0.003   0.994

Explain the output.  Go back.
```

---
